# Feasibility and Inter-rater Reliability of the Japanese Version of the Intensive Care Unit Mobility Scale

**DOI:** 10.7759/cureus.59135

**Published:** 2024-04-27

**Authors:** Daisetsu Yasumura, Hajime Katsukawa, Ryu Matsuo, Reo Kawano, Shunsuke Taito, Keibun Liu, Carol Hodgson

**Affiliations:** 1 Department of Rehabilitation, Naha City Hospital, Okinawa, JPN; 2 Department of Academic Research, Japanese Society for Early Mobilization, Tokyo, JPN; 3 Healthcare Administration and Management, Kyushu University, Fukuoka, JPN; 4 Innovation Center for Translational Research, National Center for Geriatrics and Gerontology, Aichi, JPN; 5 Department of Rehabilitation, Hiroshima University Hospital, Hiroshima, JPN; 6 Department of Academic Research, Non-Profit Organization ICU Collaboration Network (ICON), Tokyo, JPN; 7 Critical Care, Monash University, Monash, AUS; 8 Intensive Care Unit, Alfred Hospital, Melbourn, AUS

**Keywords:** the intensive care unit mobility scale, inter-rater reliability, feasibility study, physical therapy rehabilitation, early mobilization

## Abstract

Purpose

The purpose of this study was to verify the feasibility and inter-rater reliability of the Japanese version of the Intensive Care Unit Mobility Scale (IMS).

Methods

A prospective observational study was conducted at two intensive care units (ICUs) in Japan. The feasibility of the Japanese version of the IMS was assessed by 25 ICU staff (12 physical therapists and 13 nurses) using a 10-item questionnaire. Inter-rater reliability was assessed by two experienced physical therapists and two experienced nurses working with 100 ICU patients using the Japanese version of the IMS.

Results

In the questionnaire survey assessing feasibility, a high agreement rate was shown in 8 out of the 10 questions. All respondents could complete the IMS evaluation, and most respondents were able to complete the scoring of the IMS in a short time. The inter-rater reliability of the Japanese version of the IMS on the first day of physical therapy for ICU patients was 0.966 (95% CI: 9.94-9.99) for the weighted kappa coefficient and 0.985 (95% CI: 9.97-9.99) on the ICU discharge date assessment. The weighted κ coefficient showed an "almost perfect agreement" of 0.8 or higher.

Conclusion

The Japanese version of the IMS is a feasible tool with strong inter-rater reliability for the measurement of physical activity in ICU patients.

## Introduction

Critically ill patients are at risk for functional disability and decreased ability to perform activities [1-2]. Previous studies have reported that early mobilization and early rehabilitation in the intensive care unit (ICU) can improve the functional prognosis of patients undergoing intensive care management, as well as improve outcomes [3-6] and reduce medical costs [7,8]. Although severe adverse events were rare during mobilization in ICU [9], the occurrence rate of potential adverse events in intubated patients was 6.6% [10]. An accurate evaluation of a patient's mobility level is important to safely provide rehabilitation in ICU. An example of the tools for the evaluation of a patient's activity level is the Functional Status Score for the ICU (FSS-ICU), Chelsea Physical Assessment Tool (CPAx), Physical Function in ICU Test-scored (PFIT-s), and Intensive Care Unit Mobility Scale (IMS) [11]. The Intensive Care Unit Mobility Scale (IMS) is a 10-item questionnaire with an 11-point scale from 0 to 10 where 0 represents no active moving (lying in bed) and 10 represents walking at least 5 meters without assistance. Using the IMS, it is possible to evaluate a patient's mobility level with objective quantification. This scale is a useful tool for sharing information in order to evaluate ICU patients' mobility accurately. The feasibility and inter-rater reliability of the IMS for assessing activity levels in patients admitted to the ICU has been demonstrated in international studies [12]. The IMS has also been shown to have construct and predictive validity for post-ICU outcomes, including for the prediction of return home and 90-day mortality [13].

The Japanese version of the IMS was created by Japanese medical professionals [14]. The completed Japanese version was released (Appendix 1), including cross-cultural adaptation with a multidisciplinary committee [15]. However, the Japanese version of the IMS has not yet been applied in clinical practice because no studies have examined its feasibility and inter-rater reliability in ICU patients. Thus, the purpose of this study was to explore the feasibility and inter-rater reliability of the Japanese version of IMS in the ICU.

## Materials and methods

Setting

This study comprised a prospective observational study conducted at Naha City Hospital and Maebashi Red Cross Hospital, both of which have ICUs, between April and July 2018. This study was approved by the Ethical Review Committee of the Japanese Society for Early Mobilization (approval number: 2018103) and then by the Ethical Review Committees of Naha City Hospital (approval number 18004a4) and Maebashi Red Cross Hospital (approval number 30-4). All data was obtained after the date of ethical review committee approval. The need for written consent was waived as the study was observational, with no identifying data recorded. The study was divided into two components: evaluation of the feasibility of the Japanese version of the IMS using a questionnaire survey and measurement of the inter-rater reliability of the Japanese version of the IMS.

Feasibility

Nurses and physical therapists who have not experienced the evaluation using the Japanese version of the IMS were included in the feasibility study. For the evaluation of feasibility, a 10-item questionnaire to gauge medical professionals' feelings regarding the use of the Japanese version of the IMS was developed based on former research [12], with responses given in a three-option format: "Yes", "No", or "Unclear" (Appendix 2). Staff who have not experienced patient assessment and implementing plans regarding weaning and rehabilitation in the ICU were excluded. These staff members were given pre-education regarding the use of the Japanese version of the IMS for a month. The contents of pre-education included: 1) two 30-minute educational lectures explaining how to use the IMS and the significance of assessing physical activity and terms such as "tilt bed" and "active exercise" that are not used in daily clinical practice; and 2) assess ICU patients under supervision using the IMS. Pre-education was not provided according to clinical experience.

Inter-rater reliability

For the measurement of inter-rater reliability, patients' physical activity levels were assessed using the Japanese version of the IMS. Patients aged ≧18 years admitted to two ICUs during the study period were included. Patients with the following traits were excluded: 1) paralysis from central nervous system disease; 2) post-cardiopulmonary arrest; 3) patients who had lost ADL independence before hospitalization; 4) patients receiving end-of-life care. Physiotherapists and nurses with more than 10 years of experience in intensive care were selected at each hospital to evaluate the IMS. Evaluators received the same pre-educational training on measurement as in the feasibility study. Assessments were conducted twice - once during the initial mobilization session and again at ICU discharge. Each evaluator assessed the same patient once within 10 minutes of the end of the patient mobilization based on the patient's mobility. Patients who could not complete the evaluation within 10 minutes after mobilization were excluded from the analysis as missing data. The IMS grade result was recorded by each evaluator independently without consulting with other evaluators. Age, gender, severity of illness (APACHE II), disease classification (medical, surgical, trauma), ventilator status, tracheostomy status, and duration of ventilator management were also recorded.

Statistical analysis

The number of respondents for the feasibility questionnaire was set at 25. Four evaluators were selected for the examination of inter-rater reliability, and 50 patients at the two participating hospitals were selected, making a total of 100 patients. The number of subjects for the feasibility and the inter-rater reliability was estimated accurately following the previous model of study [12]. Simple tabulations were performed for the analysis of the feasibility questionnaire results.

For inter-rater reliability, simple κ coefficients, weighted κ coefficients [16], and Spearman's rank correlations among evaluators were calculated in addition to the simple tabulations of measurement results. Simple κ coefficients and weighted κ coefficients were evaluated as almost perfect agreement of 0.8 or more, high agreement of 0.6 to less than 0.8, and moderate agreement of 0.4 to less than 0.6 [17,18].

Data analysis was conducted at institutions other than the two institutions where the data was collected to ensure objectivity and avoid arbitrary processing. The data was analyzed using SAS 9.4 software (SAS Institute Inc, Cary, North Carolina).

## Results

Feasibility

Respondents to the feasibility questionnaire were comprised of 12 physiotherapists (seven Naha, five Maebashi; 11 (6.3-11.0) years of experience: median (IQR)) and 13 nurses (eight Naha, 5 Maebashi; 9.5 (5.5-12.0) years of experience) working in the ICUs of the two hospitals (Table 1). The questionnaire results are shown in Table 2. More than 80% of the respondents agreed on feasibility for eight out of the ten questions. All respondents could complete the IMS evaluation, and 92% of the respondents completed it within five minutes. To the question asking whether the Japanese version of the IMS contains any inappropriate or misleading expressions or extra steps (Question 6), 24% of respondents answered "yes". The number of nurses who answered "yes" to this question was twice that of the physical therapists.

**Table 1:** Characteristics of the medical staff PT - physical therapist; RN - registered nurse

Variable (N=25)		
Occupation background, n (%)		
PT		12 (48%)
RN		13 (52％）
Clinical experience, median (IQR)		
PT		11 (6.25 - 11.0)
RN		9.5 (5.5 - 12.0)

**Table 2:** Feasibility survey of the Japanese version of the ICU Mobility Scale (IMS-J) (N=25) IMS - Intensive Care Unit Mobility Scale; PT - physical therapist; RN - registered nurse

	Yes ％ (PT, RN)	NO ％ (PT, RN)	Unclear ％ (PT, RN)
Is the IMS-J clear and unambiguous?	96 (44, 52)	4 (4, 0)	0 (0, 0)
Did the IMS-J take less than 1 min to compete?	56 (32, 24)	36 (16, 20)	8 (0, 8)
Did the IMS-J take less than 5 min to compete?	92 (44, 48)	8 (4, 4)	0 (0, 0)
Does the IMS-J indicate what the scale is about and the overall purpose?	88 (48, 40)	4 (0, 4)	8 (0, 8)
Are there adequate definitions of the IMS-J, with examples?	84 (40, 44)	4 (0, 4)	12 (8, 4)
Are any levels of the scale irrelevant, misleading or superfluous?	24 (8, 16)	64 (32, 32)	12 (8, 4)
Are any levels of the scale offensive or otherwise inappropriate?	16 (8, 8)	80 (36, 44)	4 (4, 0)
Will respondents know the answer to the questions?	88 (40, 48)	4 (0, 4)	8 (8, 0)
Are any items unnecessary or repetitive?	12 (12, 0)	84 (32, 52)	4 (4, 0)
Is it an appropriate length?	84 (44, 40)	12 (4, 8)	4 (0, 4)

Inter-rater reliability

Two physical therapists (with 10 and 12 years of intensive care experience) and two nurses (with 11 and 15 years of intensive care experience) collected data in order to examine inter-rater reliability. A total of 453 patients were admitted to the ICUs of the two hospitals during the study period. Of these, 353 patients were excluded, and the remaining 100 patients (50 patients at each institution) were included in the analysis. The median (IQR) age of the patients was 70.0 (62.0-79.0) years (Table 3). The median of the IMS assessment results at the initial mobilization and ICU discharge was 1.0 (0-1) and 4 (1-6), respectively. 

**Table 3:** Patients' demographics and characteristics SD - standard deviation; IQR - interquartile range;  IMS - Intensive Care Unit Mobility Scale All variables had no missing data for the 100 patients.

Variables (N=100)	
Age, median years (IQR)	70.0 (62.1 - 79.0)
Female, number (%）	47 (47%)
APACHE II, median (IQR）	15.5 (10.3 - 21.0）
Reason for admission to ICU, number	
Medical	42
Surgical	47
Trauma	11
ICU duration, median days (IQR)	4.0 (3.0 - 7.0)
Mechanically ventilated, number (%)	42 (42％)
Mechanical ventilation duration, median days (IQR)	1.0 (0.0 - 3.0)
Tracheostomy present, number (%)	8 (8%)
Readmitted, number (%)	0 (0%)
The IMS assessment results, median (IQR)	
1st evaluation (the initial mobilization session)	1 (0 - 1)
2nd evaluation (ICU discharge)	4 (1 - 6)

The weighted kappa coefficient was 0.966 (95% CI: 9.94-0.99), and the Spearman rank correlation coefficient was 0.970 (9.96-0.98) on the first day of physiotherapy. The weighted kappa coefficient for the Japanese version of the IMS assessed on the day of discharge from the ICU was 0.985 (9.97-0.99), and the Spearman rank correlation coefficient was 0.999 (9.99-1.00) on the day of ICU discharge ICU (Table 4).

**Table 4:** Inter-rater reliability of the IMS-J IMS - Intensive Care Unit Mobility Scale; CI - confidence interval; ICU - intensive care unit; PT - physical therapist; RN - registered nurse

Comparison groups (PT vs. RN)	N	Kappa coefficient (95％ CI）	Weighted Kappa (95% CI)	Spearman rho (95% CI)	p-value
						1st evaluation (the initial mobilization session)	100	0.923 (0.89 to 0.98)	0.966 (0.94 to 0.99)	0.970 (0.96 to 0.98)	0.922
2nd evaluation (ICU discharge)	100	0.942 (0.89 to 0.99)	0.985 (0.97 to 0.99)	0.999 (0.99 to 1.00)	0.943

## Discussion

The Japanese version of IMS was easy to apply in intensive care environments. In our survey of 12 physical therapists and 13 nurses to estimate the feasibility of the Japanese version of the IMS, a high agreement rate was shown in 8 out of the 10 questions. In particular, positive responses were obtained for questions that asked about clarity of expression, the purpose of the evaluation, and whether it was possible to answer the evaluation questions. All respondents could complete the IMS evaluation, and most respondents were able to complete the scoring of the IMS in a short time. Based on these findings, the Japanese version of the IMS was feasible, and it is possible for medical staff working in ICUs to apply the Japanese version of the IMS in clinical practice.

However, our results showed a lower affirmative rate than the findings of previous studies for only one question. When asked about the time required for the evaluation, 92% of the respondents answered that they could complete it in a short time (five minutes), but only 56% answered that they could complete it in a very short time (one minute). In the former study for the English version of the IMS [12], 90% of the respondents were able to complete the IMS evaluation within one minute. This disparity was presumed to have arisen from the difference in culture regarding early mobilization among countries. The implementation rate in Japan is lower than in Australia aspect of mobilization in ICU [19,20]. Inexperienced staff spend time for evaluation using the IMS. Cultural differences among countries may influence the time for IMS evaluation.

As with previous studies conducted using the English version of the IMS, a low agreement rate was obtained for the question asking whether the Japanese version of the IMS contained any irrelevant or misleading expressions. A detailed analysis of the data showed that the number of nurses who answered "yes" was twice as high as the number of physical therapists, as can be seen in Table 2. Chamberlain-Salaun et al. have reported that there are differences in the understanding of technical terms among medical professions [21]. Therefore, for the IMS Japanese version to be used in clinical practice, it is essential to create educational tools with technical terms that can be understood by multiple professions.

Regarding inter-rater reliability, the weighted κ coefficient and the Spearman rank correlation coefficient all showed an "almost perfect agreement" of 0.8 or higher. The initial evaluation and the evaluation at ICU discharge also showed high agreement. Those results were better than the English version of the IMS [12]. Specific pre-education on the clinical use of the Japanese version was set in our study. Pre-educational training may affect the inter-rater reliability of the IMS. A unique feature of this study was that it assessed patients on the date of their initial physical therapy in the ICU and on the date of their discharge from the ICU when recovery of physical activity was expected. It was anticipated that IMS assessment results by the two professions would show variance at the time of discharge when patients perform higher activity. However, contrary to these predictions, the IMS assessment results at the time of discharge from the ICU showed a tendency toward inter-professional agreement. A previous study [22] reported that nurses tended to estimate patients' activity levels lower than physical therapists. In contrast to that report, the present study found a tendency for the IMS grades of the physical therapists and nurses to coincide. These results indicate strong inter-rater reliability of the Japanese version of the IMS between different professions. It is important for rehabilitation in ICUs to monitor physical activity levels among various professions. The Japanese version of the IMS may be useful for understanding the level of physical activity between different professions. In addition, clinical use of the IMS may help implement a team approach regarding mobilization for ICU patients.

Limitations

There are several limitations to this study. Firstly, the estimation for the number of evaluators and patients was not based on power analysis. The interpretation of the findings needs attention because of the risk of bias. Secondly, inter-rater reliability was assessed for evaluators with sufficient years of ICU experience. It is unclear whether similar results would be obtained for evaluators with fewer years of experience. However, inter-rater reliability was high between senior staff and junior staff in a previous study for the English version of the IMS [12]. Moreover, this study examines the feasibility and inter-rater reliability of the Japanese version of the IMS but not its validity. Thus, the validity of the Japanese version of the IMS for clinical and research use needs to be examined in the future.

## Conclusions

The feasibility and reliability of the Japanese version of IMS has been studied. High agreement rate was shown in the questionnaire for feasibility. The IMS assessment results by the two professions showed a high inter-rater reliability. The Japanese version of the IMS is a feasible tool with strong inter-rater reliability for the measurement of physical activity in ICU patients.

## Figures and Tables

**Figure 1:**
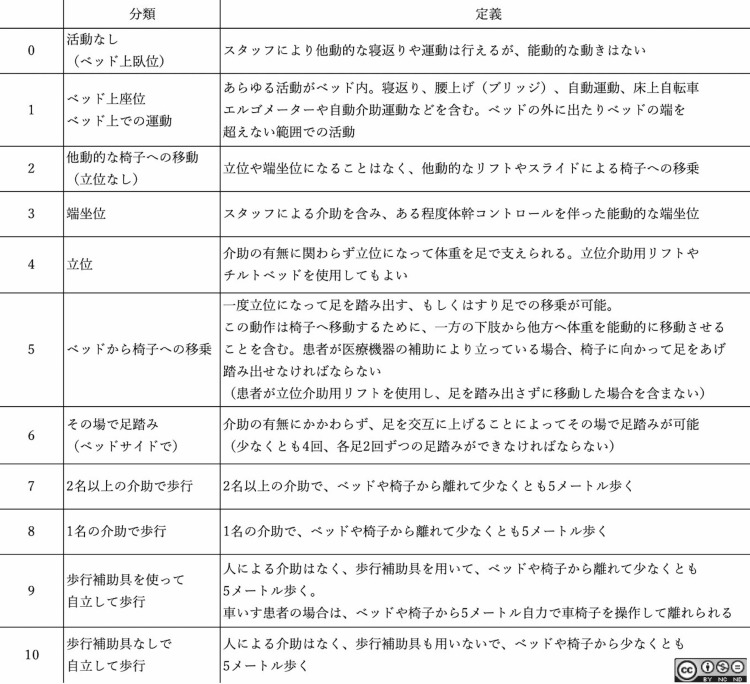
Appendix 1: the Japanese version of the IMS IMS - Intensive Care Unit Mobility Scale The Japanese version of the IMS is licensed under the Attribution– Non-Commercial-NoDerivs 4.0 International License. To view a copy of this license, visit http://creativecommons.org/licenses/by-nc-nd/4.0/

**Figure 2:**
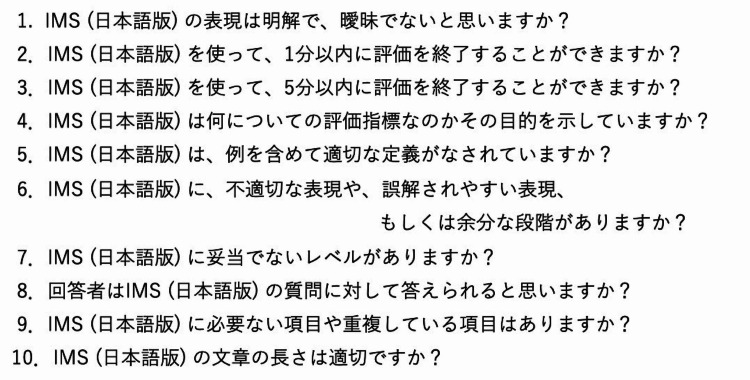
Appendix 2: survey form of the ICU Mobility Scale (IMS) IMS - Intensive Care Unit Mobility Scale
